# Elucidating physiological and biochemical alterations in giant duckweed (*Spirodela polyrhiza* L. Schleiden) under diethyl phthalate stress: insights into antioxidant defence system

**DOI:** 10.7717/peerj.8267

**Published:** 2020-01-09

**Authors:** Ritika Sharma, Rajinder Kaur

**Affiliations:** Department of Botanical and Environmental Sciences, Guru Nanak Dev University, Amritsar, Punjab, India

**Keywords:** MDA, Oxidative stress, Ecotoxicology, *Spirodela polyrhiza*, Diethyl phthalate

## Abstract

**Background:**

The emollient properties of phthalates have led to their extensive production and intense use in plastic products. Owing to their weak covalent bonding with the plastic polymers, phthalates enter into the environment during their manufacturing, processing, disposal, consequently found their way directly into water sources, soil, and sediments.

**Methods:**

The present study envisaged the toxic effects of diethyl phthalate (DEP) on physiological and biochemical attributes of *Spirodela polyrhiza*, when exposed to various concentrations of DEP (0, 10, 20, 40, 80, 100, 200, and 400 ppm) for short term exposure period of seven days.

**Results:**

Plants of *S. polyrhiza* accumulated significant amount of DEP (112 mg kg^−1^ fw) when exposed to various concentrations of DEP for seven days. Results depicted that DEP toxicity significantly (*p* ≤ 0.05) affected growth parameters and pigments in treated *S. polyrhiza* as compared to control. Further, high doses of DEP (400 ppm) caused significant decrement in carbohydrate (86%), protein (76%) and elevation in MDA content (42%). Meanwhile, DEP altered the activities of antioxidant enzymes (SOD, CAT, APX, GPX and GR) along with the induction of enhanced levels of proline, electrolyte leakage and phenolic content. Scanning electron microscopic and confocal studies also confirmed oxidative stress in plants under DEP stress.

**Conclusions:**

Present findings will help understand the accumulation, tolerance, and detoxification mechanisms of DEP by *S. polyrhiza* to counteract the effects of reactive oxygen species (ROS), along with the evaluation of environmental threat for aquatic plants in aquatic ecosystems.

## Introduction

Phthalates or phthalic acid esters (PAEs) are a group of organic compounds that are widely used as additives in many plastic products. PAEs are intended to soften and improve the flexibility of various products such as paints, toys, home furniture, synthetic fibers, and varnishes (Ventrice et al., 2013; Sun et al., 2012). There has been a tremendous increase in the production of PAEs, with an average annual production in the world was recorded to be 6 million tons per year (Niu et al., 2014). PAEs detach easily from plastic products as they are bound *via* weak vander waal interaction or hydrogen bond (Gao & Wen, 2016). Thus, PAEs easily leach out from the plastic products and enter into the surrounding environment during their large scale production and extensive use (Huang et al., 2008; Chi, 2009). Interestingly, PAEs have the potential to bioaccumulate in living organisms and due to their suspected carcinogenic, estrogenic, and teratogenic properties, PAEs have become a threat to human health and the ecosystem (Zhang et al., 2015). A considerable amount of attention has been paid to adverse effects of PAEs on human health in recent years because of their tendency to cause endocrine disruption, mutagenicity, teratogenicity, reproductive disorders, hypertension, hypospadias and malformations (Gomez-Hens & Aguilar-Caballos, 2003; Koch et al., 2005; Amin et al., 2018). Furthermore, USEPA (United States Environmental Protection Agency) classified six phthalates as priority organic pollutants, namely diethyl phthalate (DEP), dimethyl phthalate (DMP), dibutyl phthalate (DBP), butyl benzyl phthalate (BBP), di-n-octyl phthalate (DnOP) and diethylhexyl phthalate (DEHP) (USEPA, 2013; Wang et al., 2013; Zhang et al., 2016). Perusal of literature reported frequent occurrence of PAEs in aquatic and terrestrial organisms such as algae, phytoplankton, zooplankton, mice, fish, and also in human urine, blood, breast milk and saliva (Mankidy et al., 2013; Gu et al., 2017; Bai et al., 2017), leading to harmful consequences in these organisms. 

Diethyl phthalate (DEP) is also one of the most common PAEs in air, soil, and water (Wang et al., 2008; Staples, Parkerton & Peterson, 2000). It is colorless and has a faint, disagreeable odor, often used in cosmetics, and fragrances (Api, 2001). Besides this, other industrial uses include plasticizers, detergent base, aerosol sprays, herbicides, and coating of medicines (Ghorpade et al., 2002; McCarroll, 2006). Likewise other PAEs, DEP is also unable to link covalently to polymer products and readily enters into the environment. Thus, a significant amount of DEP is quite detectable in air, soil, and water that led to exposure of living organisms to this compound (Leitz et al., 2009; Langer et al., 2010). Concentration of DEP in drinking water ranges from 0.00001–0.0046 ppm, in river water at 0.00006–0.044 ppm, and in industrial wastewaters at 0.00001–0.060 ppm (ATSDR, 1995). Aquatic organisms like fish accumulated about 2 ppm of DEP in their tissues, while 1ppm of DEP was reported in oysters. Moreover, DEP in plastic packaging may incorporate into food products at concentrations of about 2–5 ppm. Also, the daily consumption of DEP by human beings has been estimated to be 4 mg based on food intake. However, annual exposure from drinking DEP contaminated water has been estimated to be minimal (0.0058 mg/year/person) (ATSDR, 1995).

The fate of DEP in the environment is also widely explored in plants, as DEP act as a stressor to plants. Previous literature data reported the adverse effects of DEP on plants as it inhibited seed germination, root, and shoot elongation (Saarma et al., 2003; Xuan et al., 2006). Saarma et al. (2003) observed the role of DEP in retarding the growth of radish (*Raphanus sativus*). Further experiments by the authors, dealing with the study of in vitro protein labeling coupled with two-dimensional gel electrophoresis revealed that heat shock proteins (HSPs) were not affected by DEP (Saarma et al., 2003), although some heat shock proteins act as an indicator of DEP stress. Previous reports suggested that DEP may act as phytotoxins (Xuan et al., 2006). Cheng (2012) observed acute toxic effects of DEP on greater duckweed at concentrations ranged from 0 to 2 mM. However, much attention has been given to oxidative effects on animals and terrestrial plants in literature but very little information is available regarding physiological and biochemical perturbations caused by DEP on aquatic plants, as it is still under investigation. So, the present study envisaged the role of DEP in retarding the growth of *Spirodela polyrhiza* and alterations of various biochemical indices by triggering the generation of reactive oxygen species (ROS) during DEP mediated oxidative stress. Oxidative stress refers to a disproportioned cellular redox reaction that causes DNA disruption, membrane damage, enhancement in lipid peroxidation and inhibition of protein synthesis (Ruley, Sharma & Sahi, 2004; Sharma & Kaur, 2018). To cope with the oxidative stress and to counteract the harmful effects of ROS, plants have evolved a complex network of antioxidant defence mechanism. This involves a plethora of antioxidant enzymes such as superoxide dismutase (SOD), catalase (CAT), ascorbate peroxidase (APX), guaiacol peroxidase (GPX), and glutathione reductase (GR).

*Spirodela polyrhiza*, commonly known as duckweed, was employed as a model plant in this present investigation to evaluate the phytotoxicity of DEP and its accumulation potential as it is very easy to culture in lab conditions and very sensitive to heavy metals and pollutants (Appenroth et al., 2010). Thus, the present study was conducted to explore the DEP accumulation potential of *S. polyrhiza* interference with various biochemical parameters, namely chlorophyll, anthocyanin, protein, carbohydrate, MDA, phenol, electrolyte leakage, and proline content, and also its detoxification potential by activation of antioxidant defence system (SOD, CAT, APX, GPX and GR). Scanning electron and confocal micrographs also revealed oxidative stress in plants under DEP stress. This study will help explore the underlying mechanisms of DEP toxicity to *S. polyrhiza* by correlating the DEP inference with plant growth and oxidative stress indicators and to figure out oxidative damage due to exposure of DEP.

## Materials and Methods

### Experimental material, plant growth conditions and treatments

Plants of *Spirodela polyrhiza* were collected from Sewage Treatment Plant (STP) of Guru Nanak Dev University, Amritsar, where it flourishes in abundance in the treated chamber. Plant material was cleaned properly with distilled water and then acclimatized for a week in 3% Hoagland nutrient medium in a seed germinator under controlled conditions (temperature: 25  ± 2 °C; light intensity: 115 µmol m^2^ s^−1^ light/dark cycle: 16/8 h). The Hogland nutrient medium comprised of macronutrients (KNO_3_, CaNO_3_.4H_2_O, Iron, MgSO_4_.7H_2_O, NH_4_NO_3_), micro nutrients (H_3_BO_3_, MnCl_2_.4H_2_O, ZnSO_4_.7H_2_O, CuSO_4_.5H_2_O, Na_2_MoO_4_.2H_2_O, Fe-EDTA) and phosphate (KH_2_PO_4_) mixed in 1000 ml Millipore water. After acclimatization period, healthy fronds of *S. polyrhiza* were inoculated in Petri plates containing 100 ml of Hoagland nutrient medium, where plants without having DEP added in the medium served as control. *S. polyrhiza* were exposed with different concentrations of DEP (0, 10, 20, 40, 80, 100, 200 and 400 ppm) for seven days in triplicates.

Diethyl phthalate (DEP) (99.0% purity, CAS: 84-66-2) was purchased from Hi-Media, Mumbai (India). Other chemicals used as ingredients of the Hoagland nutrient medium (3%) were of analytical grade. Stock solution of DEP was prepared using 1 ml of ethanol, 2-3 drops of Tween-20 and distilled water in the required proportion to obtain the solubility of solution (Chen et al., 2011). Further, required concentrations for treatment were prepared by diluting a stock solution of phthalate in the Hoagland nutrient medium. The plants were harvested after seven days and then stored at −80 °C, till analyzed for various growth and biochemical parameters.

### Analysis of accumulated DEP content in *S. polyrhiza*

Accumulated DEP content in *S. polyrhiza* was analyzed by the ultrasonication method of Ma et al. (2013) with some modifications. The plant sample (0.5 g) was homogenized in 20 ml acetone (HPLC grade). Extract was collected and sonicated for 30 min. 20 ml hexane (HPLC grade) was added and further sonicated for the next 30 min. Filteration of the extract was done using Whatman no. 42 filter paper and the extract was evaporated in a rotary evaporator until the volume reduced to 5 ml. Hexane (2–3 ml) was added in the remaining extract and again evaporated to 1 ml. Finally, 4 ml acetonitrile (ACN) was added and the extract was filtered using a syringe filter with dimension 0.22 µm before analysis. Analysis of accumulated DEP content in plant was determined using reverse phase-high performance liquid chromatography (RP-HPLC) purchased from Shimadzu (Japan). The method validation for phthalates analysis was carried out for our present investigation using normalized guidelines of the International Conference on Harmonisation of Technical Requirement for Registration of Pharmaceutical for Human Use (ICH, 2005).

### Determination of growth parameters

Harvested plant materials were analyzed for various growth and biochemical parameters. Before weighing, plants were cleaned properly and excess moisture was dried by placing the fronds between the two folds of filter paper and pressed gently. Measurement of the dry weight of the plant material was taken after oven drying at 105 °C for the first 20 min and then at 80 °C for next 48 h to obtain constant dry weight. Percentage change in fresh weight and dry weight to fresh weight ratio was then calculated (Sharma & Kaur, 2017).

### Pigment analysis

Chlorophyll content was estimated according to the protocol given by Arnon (1949). Carotenoid content was measured by the method of Lichtenthaler & Wellburn (1983). Anthocyanin content was determined by the protocol given by Mancinelli (1984).

### Determination of protein and carbohydrate content

The measurement of soluble protein content was done by homogenizing the plant material in potassium phosphate buffer (pH = 7). Extract was centrifuged and protein content was determined according to the Bradford method (1976). For carbohydrate determination, the anthrone method was used (Yemm & Willis, 1954).

### Measurement of electrolyte leakage and malondialdehyde content

Electrolyte leakage was determined by the method of Dionisio-Sese & Tobita (1998). Plant material (0.2 g) was cut into pieces and immersed in test tubes with distilled water (10 ml). Then incubated at 40 °C for 2 h. Initial electrical conductivity (EC1) was recorded using a conductivity meter. Samples were autoclaved and final electrical conductivity was recorded (EC2). Percent of electrolyte leakage was calculated as:

\begin{eqnarray*}& & \mathrm{Electrolyte} \mathrm{leakage} (\text{%})= \frac{\mathrm{ECI}}{\mathrm{EC}2} \times 100. \end{eqnarray*}

MDA content was measured according to the method of Heath & Packer (1968). Plant material was homogenized in 0.1% trichloroacetic acid (TCA) and centrifuged for 10 min. Then reaction mixture comprising supernatant, 0.5% thiobarbituric acid (TBA) and 20% TCA was heated at 95 °C in a boiling water bath for 30 min. After centrifugation at 10,000 rpm, the absorbance of the supernatant was read at 532 nm and non-specific absorption was corrected by subtracting the absorbance value observed at 600 nm. The concentration was expressed µmol g^−1^ fw.

### Determination of phenolic and proline content

Total phenolic content was assayed by the protocol of Singleton & Rossi (1965). Absorbance was read at 765 nm and results were expressed as mg g^−1^ fw. Proline content in the plant was done by following the method described by Bates, Waldren & Teare (1973). Absorbance was read at 520 nm. Measurement of proline content was expressed in µmol g^−1^ tissue. L-proline was used as a standard.

### Antioxidant enzymatic assays

Plant sample (1 g) was homogenized in 3 ml ice-cold potassium phosphate buffer (0.1 M) (pH = 7). The homogenate was centrifuged and the supernatant was used for the analysis of enzymatic activities (SOD, CAT, APX, GPX, and GR). Superoxide dismutase (SOD) (EC 1.15.1.1) activity was evaluated by the method given by Kono, Takahashi & Asada (1979). Catalase (CAT) (EC 1.11.1.6) activity was assessed by the method given by Aebi (1974). Ascorbate peroxidase (APX) (EC 1.11.1.11) activity was analyzed by following the protocol of Nakano & Asada (1981). Guaiacol peroxidase (GPX) (EC 1.11.1.7) activity was done according to the method given by Pütter (1974). Glutathione reductase (GR) (EC. 1.6.4.2) activity was determined by the protocol of Carlberg & Mannervik (1985).

### Scanning electron microscopic (SEM) studies

The plant surface was imaged at high resolution using scanning electron microscopy. Scanning electron microscopy was done according to the method of Liu et al. (2000). The stomatal response of control and DEP treated fronds of *S. polyrhiza* was observed by using a scanning electron microscope (Carl Zeiss-EvoLS 10). Samples were prepared by fixing leaf samples in 2.5% glutaraldehyde prepared in 0.1 M potassium phosphate buffer and kept overnight. Leaf samples were washed with distilled water and dehydrated in different ethanol series (30%, 50%, 70%, and 90%) for 20 min. Adaxial surface of leaves were then placed on spherical metal stubs, fixed using adhesive tape and silver-coated in sputter coater instrument. The surface features of leaves were viewed under a scanning electron microscope at a voltage of 15 kV and stomata were viewed under the resolution of 500–4,000 nm.

### Confocal laser scanning microscopic (CLSM) studies

For confocal microscopy, roots of control and treated plant samples were washed with distilled water and then treated with different confocal dyes i.e., Propidium iodide (PI), dichlorofluorescein diacetate (H2DCFDA) and monochlorobimane (MCB) respectively in dark for 10 min separately for studying cell viability, detection of ROS and GSH levels occurred during stress in plants. Roots were treated with different fluorescent dyes during dark conditions. After treatment, roots were washed thoroughly and placed on glass slide over a drop of water to prevent dehydration and covered with cover slip. For propidium iodide, He-Ne gas laser was used to excite the electrons at a wavelength of 535 nm, multiline argon gas laser was used for 2, 7 dichloroflurescein (DCF) to excite the electrons at the wavelength of 488 nm and for monochlorobimane excitation wavelength of 380 nm was used.

### Statistical analysis

All the experiments were performed in triplicates and results were expressed as the mean  ± standard error. The data were subjected to one-way analysis of variance (ANOVA) for assessing the effect of DEP on *S. polyrhiza*. Tukey’s post hoc multiple comparison test was done at 0.05 level of significance for the comparisons against control values. 

## Results

### Analysis of accumulated DEP content in *S. polyrhiza*

**Figure 1:**
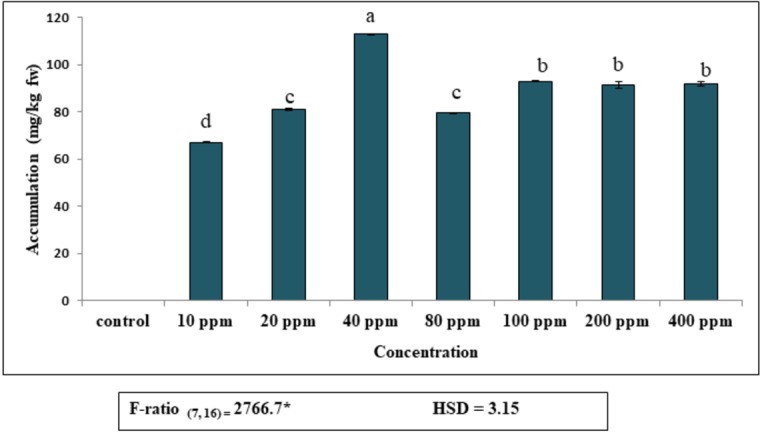
Accumulation of diethyl phthalate (DEP) by *S. polyrhiza*. Results are presented as Mean ± SE, *n* = 3, one-way ANOVA, Tukey’s HSD. *Significant at *p* ≤ 0.05. Control showed no DEP detection. Same letter means does not significantly differ at *p* ≤ 0.05.

Accumulation of DEP by fronds of *S. polyrhiza* was initially rapid and then dropped gradually, becoming almost constant at higher concentrations (Fig. 1). Plants exposed to 40 ppm DEP accumulated the highest amount (112.8 mg kg^−1^ fw) after seven days of culture. Data revealed that DEP in the observed concentrations (10–400 ppm), the maximum percentage of accumulation was 68%, as compared to control and then decreased. This implies that *S. polyrhiza* is efficient in removing this much amount of DEP from low-level DEP-contaminated water for seven days. The amount of DEP accumulated by the plant becomes almost constant at higher concentrations of 200 ppm (91 mg kg^−1^ fw) and 400 ppm (91.7 mg kg^−1^ fw).

### Exposure to increased concentrations of DEP induced growth inhibition

Abiotic or biotic stress in plants bring changes in the growth rate and other metabolic processes. We observed that *S. polyrhiza* showed significant (*p* ≤ 0.05) reduction in plant growth with increasing concentrations of DEP after 7 days of exposure (Table 1). Most phytotoxic effects of DEP was observed at a concentration of 400 ppm. Notably, the maximum percent decrease in fresh weight was found 54% and dry to fresh weight ratio was found 86% at highest concentration of 400 ppm of DEP respectively as compared to control (Table 1).

**Table 1:** Effect of diethyl phthalate (DEP) on growth parameters of *S. polyrhiza*. Results are presented as Mean ± SE, *n* = 3, one-way ANOVA, Tukeys HSD.

**Concentrations (ppm)**	**Fresh weight (% age)**	**Dry weight (g)**/ **Fresh weight (g)**
Control	0.80 ± 0.41e	0.34 ± 0.01a
10	6.60 ± 0.42de	0.29 ± 0.01b
20	12.40 ± 1.83d	0.28 ± 0.01bc
40	18.46 ± 0.67c	0.27 ± 0.007bc
80	23.40 ± 1.24c	0.22 ± 0.01c
100	44.40 ± 1.85b	0.10 ± 0.006d
200	49.73 ± 1.23ab	0.041 ± 0.003e
400	54.60 ± 0.75a	0.045 ± 0.001e
*F*-ratio (7, 16)	301.9^*^	168.8^*^
HSD	5.81	0.045

**Notes.**

* Significant at *p* ≤ 0.05. Same letter means does not significantly differ at *p* ≤ 0.05.

### DEP induced alterations in photosynthetic pigments

The present results demonstrated that *S. polyrhiza* exposed to DEP exhibited significant reduction (*p* ≤ 0.05) in photosynthetic pigment contents during seven days culture at concentrations ranging from 10 to 400 ppm of DEP as compared to controls (Table 2). Also, treated fronds showed marked chlorosis as significant decrease in chlorophyll and carotenoid content was observed after seven days of the exposure period. As shown in Table 2, chl a content exhibited maximum decline at 40 ppm (50%), whereas, maximum decline in chl b, total chl, and carotenoid content was found to be 63, 55 and 45% at 400 ppm concentration of DEP with respect to the control. However, acceleration in the accumulation of anthocyanin pigments in fronds of *S. polyrhiza* was recorded maximum as 38% at 200 ppm DEP concentration after seven days of culture.

**Table 2:** Effect of diethyl phthalate (DEP) on chl a, chl b, total chlorophyll, carotenoids and anthocyanin pigment in *S. polyrhiza*. Results are presented as Mean ± SE, *n* = 3, one-way ANOVA, Tukeys HSD.

**Concentrations (ppm)**	**chl a (µg/ml)**	**chl b (µg/ml)**	**Total chl (µg/ml)**	**Carotenoids (µg/ml)**	**Anthocyanin(OD/gfw)**
Control	9.65 ± 0.14a	15.34 ± 1.12ab	24.99 ± 1.10ab	4.28 ± 0.05a	0.31 ± 0.034a
10	9.66 ± 0.13a	17.18 ± 0.05a	26.83 ± 0.09a	4.41 ± 0.07a	0.32 ± 0.01a
20	8.58 ± 0.18b	13.36 ± 2.10ab	21.95 ± 2.26bc	3.97 ± 0.15a	0.34 ± 0.06a
40	4.81 ± 0.03f	13.91 ± 0.10ab	18.73 ± 0.06c	3.11 ± 0.01b	0.34 ± 0.01a
80	7.72 ± 0.11c	14.58 ± 0.99ab	22.30 ± 0.87abc	2.97 ± 0.1bc	0.38 ± 0.04a
100	6.63 ± 0.06d	11.37 ± 0.19bc	18.01 ± 0.18cd	2.43 ± 0.04cd	0.42 ± 0.08a
200	6.18 ± 0.17d	7.41 ± 0.26cd	13.59 ± 0.33de	2.48 ± 0.06d	0.43 ± 0.01a
400	5.52 ± 0.06e	5.61 ± 0.53d	11.13 ± 0.49e	2.34 ± 0.18d	0.41 ± 0.04a
*F*-ratio (7, 16)	214.9^*^	17.92^*^	31.10^*^	71.18^*^	0.95
HSD	0.617	4.61	4.75	0.49	0.23

**Notes.**

* Significant at *p* ≤ 0.05. Same letter means does not significantly differ at *p* ≤ 0.05.

### DEP causes reduction in protein, carbohydrate content and enhanced malondialdehyde content

DEP at higher doses (400 ppm) was characterized by an inhibitory influence on protein and carbohydrate content in a dose-dependent manner (Table 3). Therefore, the significant decrease (*p* ≤ 0.05) by 76% in protein content was observed in DEP treated fronds at 400 ppm concentratiom as compared to untreated fronds. Similarly, carbohydrate content in DEP treated fronds also showed a decreasing trend with a maximum decrease of 86% was found at the same concentration of DEP (400 ppm) as compared to control, implying destructive effect of DEP on carbohydrates level in fronds of *S. polyrhiza*. However, MDA content increased proportionally with increased DEP concentrations and the maximum increase by 42% was observed at 400 ppm after seven days exposure period (Table 3).

**Table 3:** Effect of diethyl phthalate (DEP) on carbohydrate, protein, MDA, total phenolic content and electrolyte leakage in *S. polyrhiza*. Results are presented as Mean ± SE, *n* = 3, one-way ANOVA, Tukeys HSD.

**Concentrations (ppm)**	**Carbohydrates (mg/gfw)**	**Proteins(mg/gfw)**	**MDA content(µmol/gfw)**	**Total phenolic content(mg/gfw)**	**Proline(µmoles/g tissue)**	**Electrolyte leakage (% age)**
Control	83.89 ± 4.97a	0.55 ± 0.47a	12.17 ± 1.99ab	0.51 ± 0.16a	10.53 ± 4.72ab	56.09 ± 0.32c
10	51.79 ± 4.60ab	0.41 ± 2.30ab	14.53 ± 0.94ab	0.60 ± 0.05a	20.56 ± 5.55ab	57.59 ± 5.54c
20	41.89 ± 2.62bc	0.23 ± 4.02b	14.15 ± 1.01ab	0.80 ± 0.07a	24.14 ± 1.08ab	55.45 ± 1.32c
40	41.99 ± 16.4bc	0.24 ± 0.60b	16.12 ± 0.48ab	0.54 ± 0.11a	27.85 ± 6.31ab	77.03 ± 1.82b
80	28.69 ± 1.90bc	0.24 ± 1.18b	16.38 ± 0.07ab	0.43 ± 0.10a	21.98 ± 4.94ab	91.22 ± 1.19a
100	12.89 ± 4.21c	0.16 ± 3.24b	16.51 ± 0.90ab	0.54 ± 0.10a	31.33 ± 4.84ab	92.90 ± 1.15a
200	17.09 ± 6.90c	0.17 ± 1.78b	17.41 ± 0.39a	0.51 ± 0.09a	38.43 ± 1.70a	92.57 ± 0.09a
400	10.99 ± 4.34c	0.13 ± 2.10b	17.33 ± 0.73a	0.62 ± 0.16a	38.84 ± 1.60a	95.45 ± 2.09a
*F*-ratio (7, 16)	12.11^*^	5.78^*^	3.44^*^	0.90	4.90^*^	61.43^*^
HSD	34.39	0.28	4.78	0.57	21.06	11.38

**Notes.**

* Significant at *p* ≤ 0.05. Same letter means does not significantly differ at *p* ≤ 0.05.

### DEP increases phenolic, proline content and electrolyte leakage

A significant increase in phenolic content upto 20 ppm DEP concentration was observed, with the maximum accumulation of 60%, which then decreased at higher concentration. DEP stimulated moderate accumulation of phenols. Meanwhile, the application of DEP at 400 ppm provoked a high accumulation of proline content, leading to maximum accumulation of 264% and 268% at 200 ppm and 400 ppm respectively during seven days of the experiment. Moreover, DEP exposure also triggered the increase in leakage of electrolytes with a maximum leakage of 95% at 400 ppm concentration (Table 3).

### Exposure to increased concentrations of DEP altered antioxidant enzyme activities

**Figure 2:**
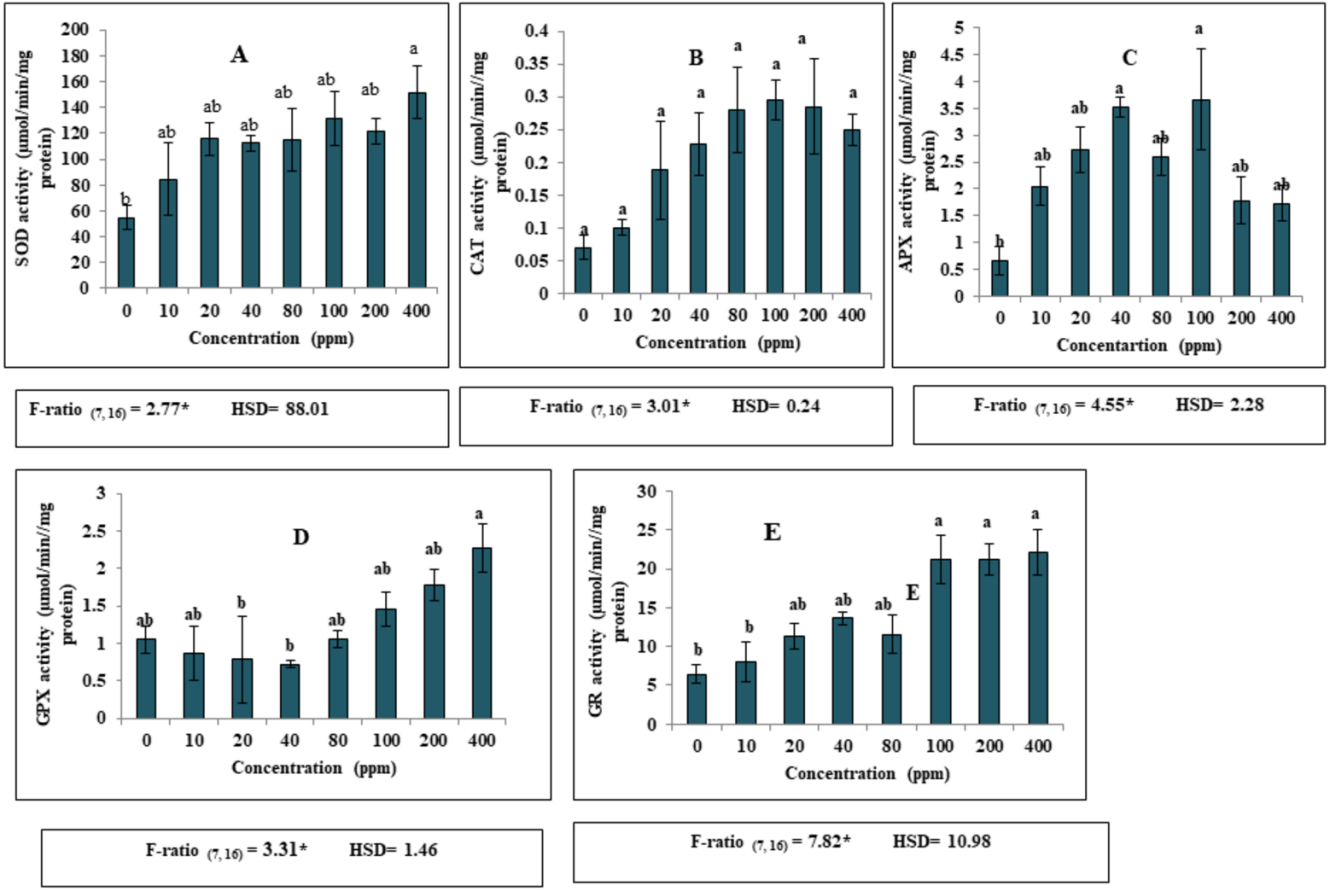
Effect of DEP stress on antioxidant enzymatic activities of *S. polyrhiza*. Results are presented as Mean ± SE, *n* = 3, one-way ANOVA, Tukey’s HSD. *Significant at ≤0.05. (A) SOD activity, (B) CAT activity, (C) APX activity, (D) GPX activity, (E) GR activity. *Significant at *p* ≤ 0.05. Same lowercase letter indicates no significant differences at *p* ≤ 0.05.

Apart from the effect of DEP on growth and biochemical parameters, DEP stress led to significant alterations in the antioxidant defence system in *S. polyrhiza*. A significant increase (*p* ≤ 0.05) in the activities of various antioxidant enzymes (SOD, CAT, APX, GPX and GR) was recorded in the fronds of *S. polyrhiza* when treated with elevated levels of DEP as compared to the non-stressed control fronds. Our present investigation revealed that 400 ppm DEP in nutrient medium resulted in high SOD activity (Fig. 2). Notably, higher activity of SOD was found to be 176% as compared to control (Fig. 2A). Likewise, CAT activity showed a significant increase (*p* ≤ 0.05) upto 100 ppm and then showed a slight decrease at 200 and 400 ppm DEP concentration as compared to control. Maximum enhancement in the activity was found to be 327% at 100 ppm concentration (Fig. 2B). Similarly, in the case of APX, a significant increase (*p* ≤ 0.05) in the activity was observed upto 100 ppm DEP concentration and then activity become almost constant at high concentrations. The highest percentage increase (452%) in the enzyme activity was recorded at 100 ppm as compared to control (Fig. 2C). GPX activity showed a significant decrease upto 40 ppm DEP concentration and then started increasing at proceeding concentrations forming an almost U-shaped pattern. A maximum activity of 53% was observed at 400 ppm DEP concentration as compared to control (Fig. 2D). A remarkable increase in GR activity recorded at all the concentrations and followed a dose-dependent trend. The maximum increase of 243% was observed in treated fronds of *S. polyrhiza* at 400 ppm concentration of DEP (Fig. 2E).

### Effect of DEP on stomata

**Figure 3:**
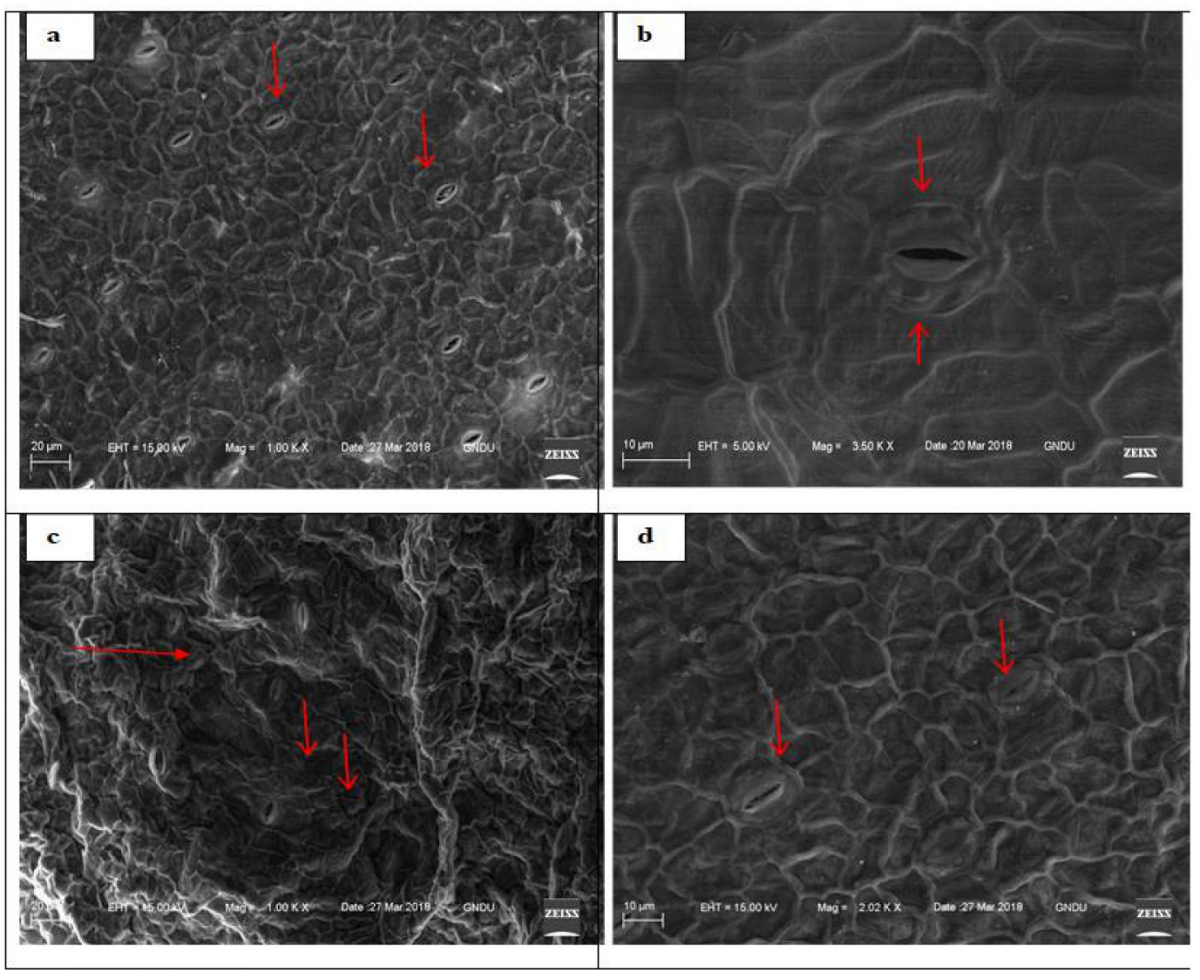
Scanning electron micrographs of adaxial surface of *S. polyrhiza* and arrows showing stomata. Untreated frond (0 ppm) (A–B) and treated (400 ppm) frond (C–D).

Scanning electron micrographs showed the marked influence of DEP on stomatal movements and morphology of stressed fronds of *S. polyrhiza* over unstressed ones. Stomata of control fronds were open, while stomata of treated fronds were mostly closed. Various other cellular changes like deformed cell shapes and collapsed cells were prominent in treated fronds as compared to untreated fronds through SEM imaging that were observed (Fig. 3).

### Exposure to increased concentrations of DEP decreased cell viability, elevated ROS and GSH levels

**Figure 4:**
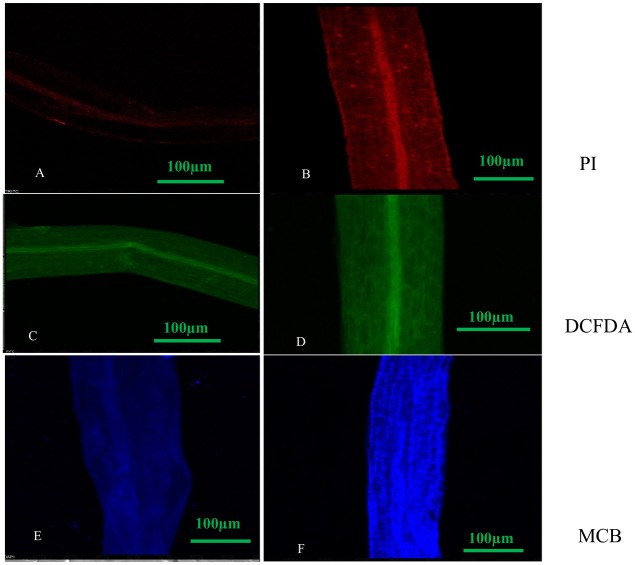
Confocal micrographs of control and DEP treated root samples of *S. polyrhiza* treated with different dyes: PI (propidium iodide), H2DCFDA (dichlorofluorosceindiacetate) and MCB (monochlorobimane). Scale bar = 100 µm. (A–F), where (A) control (PI stained), (B) treated (PI stained), (C) control (DCFDA stained), (D) treated (DCFDA stained), (E) control (MCB stained), (F) treated (DCFDA stained).

We observed that PI treated roots under DEP exposure showed more red fluorescence, indicating dead cells as compared to control with less or no fluorescence. Green fluorescence emission of DCFDA dye treated roots of plant under DEP exposure confirmed the presence of increased reactive oxygen species during oxidative stress, whereas blue fluorescence emission of MCB dye treated roots revealed enhanced GSH (glutathione) levels during oxidative stress (Fig. 4) over control roots.

## Discussion

Plants cannot escape undesired changes in the environment due to their sessile nature. Exposure of pollutants triggers series of physiological, biochemical and cellular changes, which play a pivotal role in enhancing the tolerance ability of plants and to cope with harmful consequences (Vara Prasad & De Oliveira Freitas, 2003). DEP accumulation in *S. polyrhiza* led to considerable alterations in physiological and biochemical parameters. The treated plant showed DEP accumulation in a dose-dependent manner (Fig. 1). The maximum accumulation was found at 40 ppm concentration which then decreased at 80 ppm and then became constant with preceding concentrations. However, the accumulation ability of plants decreased with an increase in dose, probably due to the toxic effects of DEP on *S. polyrhiza* (Sharma & Kaur, 2019b). Thus, the present study demonstrated that high DEP concentrations were toxic to these plants. In this context, our results are in good agreement with the dose-dependent uptake of DBP (dibutyl phthalate) by Chinese cabbage from hydroponic culture (Liao, Yen & Wang, 2009). Similar results of DBP accumulation were found in Bok choy plant (Liao, Yen & Wang, 2006). In addition, bioaccumulation potential of plant also depends upon several other environmental constraints such as temperature, pH, light, presence of other metals and anions, oxygen level and chelators (John et al., 2012). Fascinatingly, uptake of organic compounds by this free-floating macrophyte is driven by the simple process of diffusion and then enters into a leaf as solutions (Dhir, 2013). As plants are deprived of specific transporters for the transport of these organic compounds, so their movement into the plants depends on their physicochemical properties such as hydrophobicity, aqueous solubility, polarity, and molecular weight of the organic contaminant (Murray et al., 2012; Dhir, 2013). As DEP has a log octanol-water partition coefficient (log Kow) of 2.47 and a water solubility of 1100 ppm, it is reported to have toxic effects on aquatic life (Staples et al., 1997). Present analysis of DEP content in *S. polyrhiza*, revealed that accumulation increased with the increase in concentration of DEP in the medium upto some extent. This might be due to metabolic pathways for the biotransformation of organic contaminants adopted by the plant. Exposure of aquatic plants to organic compounds resulted in (i) fast uptake or sequestration of the compound into vacuoles (ii) Transformation or degradation of the compound *via* volatilization, lignification or metabolization to carbon dioxide and water (iii) Assimilation into plant tissues as non-toxic compounds (Dhir, 2013; Garrison et al., 2000).

Accumulated DEP showed deleterious effects on *S. polyrhiza* which was quite evident from decreased biomass of plants (Table 1). Herring & Bering (1988) reported similar effects of DEP on plant growth. They observed the inhibition of germination and growth of spinach and pea seedlings subjected to DEP. In this study, it is reflected by the percentage change in fresh weight, altered growth and dry to fresh weight ratio in DEP exposed fronds. Previous studies also documented that stress induced loss of turgor pressure leads to reduced mitotic activity and reduction in growth rate (Baccouch, Chaoui & Ferjani, 2001). Previous researchers also studied implications of phthalates toxicity in hampering growth of the plants. Dibutyl phthalate (DBP) and bis(2-ethylhexyl) phthalate (DEHP) were reported to inhibit shoot elongation and reduced biomass of germinating mung bean seedlings on fresh weight basis (Ting-Ting et al., 2014). Saarma et al. (2003) reported the toxic effect of DEP in retarding the growth of radish (*Raphanus sativus*). Gu et al. (2017) reported inhibition of algal growth under DBP stress.

Chlorophyll content is one of the visible indicators of stress in plants (Appenroth et al., 2010). We observed the progressive increase in DEP concentrations induced changes in photosynthetic pigments in *S. polyrhiza* (Table 2). Total chlorophyll content of the plant showed a decreasing trend upto 40 ppm as compared to control and subsequent doses of DEP. Reduction of chlorophyll content at this concentration is a consequence of either slow synthesis or rapid breakdown of chlorophyllase enzyme, suggesting photoprotection mechanism in plants *via* reducing light absorbance by decreasing chlorophyll content (Taïbi et al., 2016; Boswell, Sharma & Sahi, 2002; Gupta et al., 2009). However, the maximum reduction in chlorophyll content was observed at 400 ppm DEP concentration. Results are in good coherence with the marked reduction in photosynthetic pigments exhibited by plant species subjected to DBP and DEHP stress (Ma et al., 2015). In addition, increased anthocyanin content in this study is a part of the strategy adopted by the plant to get protected by the deleterious impacts of DEP. Anthocyanins are an important class of flavonoids that acts as pigment and DEP stress might stimulate the gene related to anthocyanin production (Sharma & Kaur, 2019a; Shan et al., 2009). Similar results were observed by Sharma & Kaur (2019b) in *S. polyrhiza* under diallyl phthalate (DAP) stress.

To gain more insights into the response of a plant to DEP stress, studies were conducted to evaluate protein, carbohydrate and MDA content in the plant., a significant decrease in soluble protein content was obtained at higher concentrations which may be probably due to protein oxidation, inhibition of protein synthesis or up-regulation of genes associated with degradation of proteins (Sytar et al., 2013). Protein degradation as a consequence of exposure to various environmental contaminants have been explored in many aquatic plants (Vara Prasad & De Oliveira Freitas, 2003; Hou et al., 2007). A similar decreasing trend was observed in *S. polyrhiza* when exposed to DBP and BBP for a period of 7 and 15 days (Kaur et al., 2017). This phytotoxic effect of DEP on *S. polyrhiza* in plant culture might be due to its role in inducing DNA damage and nucleic acid degradation. Also, DEP is characterized by an inhibitory effect on carbohydrate content in *S. polyrhiza* (Table 3). These are building substances for plants that provide energy to carry out metabolic and cellular activities. To confer osmotic adjustment to plant, stress triggers the release of monomeric forms (e.g., glucose, fructose) from polymeric forms like starch and fructans (carbohydrates) (56–57) (Sharma & Kaur, 2017; Kumari & Kaur, 2019). Reduced carbohydrate content has also coincided with enhanced degradation of photosynthetic pigments. Our results are also in coherence with those of a study conducted by Kaur et al. (2017), who reported a significant reduction in carbohydrate content in *S. polyrhiza* under a high concentration of BBP and DBP. Moreover, the stimulating influence of DEP on malondialdehyde content in this observation, is a biomarker, revealing that fronds encountered increased oxidative burst. MDA content is a result of lipid peroxidation and enhanced degradation of polyunsaturated fatty acids (PUFA) under stressful conditions (Anjum et al., 2011). It is plausible that the enhanced MDA content revealed a protective mechanism adopted by the plant for survival and tolerating DEP induced stress. Results are in agreement with those observed in mung beans under the exposure of DBP and DEHP stress (Ting-Ting et al., 2014).

Besides, to maintain osmotic balance and to confer DEP tolerance potential, fronds of *S. polyrhiza* tend to accumulate osmoprotectants (Slama et al., 2015). According to Ashraf & Foolad (2007), abiotic stress triggers proline accumulation in the cytosol and plays a pivotal role in osmotic adjustment. Importantly, the results of the present investigation also revealed enhanced accumulation of proline (Table 3). Accumulation of proline attributed to the scavenging of free radicals and to protect cell membranes. This also corroborates a dynamic relationship between increased lipid peroxidation and the proline accumulation under DEP stress. The current study is also supported by earlier studies on the impact of phthalates on *Hordeum vulgare* and cucumber seedlings (Kumari et al., 2018; Ting-Ting et al., 2014). Increased level of proline level does not indicate any sequesteration but formation of free radicals. A significant enhancement in the proline content was also observed under the exposure of BBP in water celery (Chen et al., 2011). Zhang et al. (2016) also observed augmentation in the proline content in *Cucumis sativus* under DMP stress. Increment in proline synthesis is mainly due to inhibition of oxidation of proline (Wang et al., 2007). Also, membrane damage correlated well with increased leakage of electrolytes, validated by present results of this study which showed an increase in percent leakage of electrolytes in a dose-dependent manner. Though leakage of ions showed a very slight increase (almost constant) upto 20 ppm (Table 3) as compared to control which might be due to tolerance ability of plant upto this concentration, followed by increased leakage at the other treatments with maximum leakage found at 400 ppm concentration. Probably, DEP induced cellular toxicity disrupted plant membrane resulting in increased leakage of the ions. The present investigation confirms the result of previous studies on aquatic plants (Upadhyay, 2014; Lu et al., 2011). Apart from this, DEP stimulated moderate accumulation of phenols in *S. polyrhiza* which may be due to the activation of acetate and hexose-monophosphate pathway accompanied by releasing bound phenols in stressed plant cells (Lee & Lee, 2010). Phenols have high tendency to chelate due to the presence of hydroxyl and carboxyl groups in their structure. Díaz et al. (2001) reported accumulation of phenols in *Phaseolus vulgaris* under Cu stress due to the stimulation of CHS (Chalcone synthase) and PAL (phenyalanine ammonia-lyase) activity in plant.

Generation of reactive oxygen species (ROS) is another harmful consequence of DEP accumulation by *S. polyrhiza*. According to Mittler et al. (2004), there should be equilibrium between ROS accumulation and ROS scavenging in plants. ROS, being unstable and highly reactive promptly interact with nucleic acids, pigments, lipids, proteins, causing damaging effects. Since the plethora of (ROS) can overwhelm host antioxidants and trigger oxidative stress. There are mechanisms associated to overcome the damage from free radical generation in living organisms which include the activities of antioxidative enzymes such as superoxide dismutase (SOD), catalase (CAT), ascorbate peroxidase (APX), guaiacol peroxidase (GPX) and glutathione reductase (GR) and non-enzymes such as glutathione (GSH) tocopherol (VE) and ascorbate (VC) (Bi et al., 2018). Similarly, to counterbalance the effects of reactive oxygen species (ROS), generated during stressful conditions, activation of the antioxidative defense system of the plant is critically important for the understanding of tolerance mechanisms adopted by the plant under stress. Plants have a well intricated and efficient enzymatic antioxidant defense system which includes SOD, CAT, GPX, APX, and GR. Antioxidants form a class of compounds that protects cells from oxidative damage. Enhanced or declined levels of antioxidants are correlated with increased or decreased stress tolerance of plants. Coordination of the enzymatic antioxidants helps in alleviating ROS levels (Mittler et al., 2004). Among them, SOD represents as the primary line of defence against ROS-induced severities by catalyzing the removal of O_2_ and dismutating it into O_2_^−^ and H_2_O_2_ (Gill & Tuteja, 2010; Sharma & Kaur, 2018). There are three types of SOD present in plants which include Cu-Zn SOD (cytosolic and chloroplastic), mitochondrial Mn-SOD and the chloroplastic Fe-SOD. The present results revealed that SOD activity significantly elevated in DEP-treated fronds. The extent of phytotoxic effects can be predicted by the stimulation of SOD activity at higher doses (Fig. 2). Our results coincide with the earlier reports of Ting-Ting et al. (2014) who reported increased SOD activity in germinating mung bean seedlings when subjected to DBP and DEHP. Findings suggested that the subsequent increase in the activity of SOD might be helpful in effective scavenging of O_2_^−^ to protect *S. polyrhiza* from the toxic effects of phthalates. Probably, increased activity of SOD may also be attributable to *de novo* synthesis of SOD related proteins (Song et al., 2006).

Ascorbate peroxidases (APX) is a key enzyme in the ascorbate-glutathione (ASC-GSH) cycle and removes excess H_2_O_2_ produced in the chloroplast and cytosol (Gill & Tuteja, 2010). Also, APX is considered as housekeeping protein in cytosol and chloroplast of plants and ascorbate is the substrate of this enzyme. Enhancement in APX activity was observed in this investigation. The initial stimulation of APX activity upto 100 ppm may be explained by the removal of during DEP mediated oxidative stress, as APX showed higher affinity for peroxides and dismutates H_2_O_2_ to H_2_O. Increased APX activity in response to higher various environmental constraints such as drought, salinity, and higher doses of pesticides has been well documented (Sharma & Dubey, 2005; Maheshwari & Dubey, 2009; Hefny & Abdel-Kader, 2009).

Though APX and CAT performs the similar function of scavenging H_2_O_2_, however, on the basis of their affinities (different *K*_*m*_ values), APX modulates H_2_O_2_, whereas, CAT removes excessive H_2_O_2_ (Mittler, 2002). In this present observation, DEP stimulated increment in CAT activity, maximum at 100 ppm concentration. Decrement at higher doses indicated that fronds of *S. polyrhiza* can tolerate lower concentrations of DEP upto 100 ppm, while at higher doses, the intensity of stress become so high that it leads to plant death. Moreover, imbalance in the dynamic equilibrium existing between ROS generation and its scavenging by antioxidant enzymes attributes to damaging effects on plants. A similar trend was observed by other workers in their studies dealing with the impact of phthalates on plants (Zhang et al., 2016; Zhang et al., 2014). Literature data reported increased CAT activity in *S. polyrhiza and Lemna minor* under DBP stress (Huang et al., 2006). Furthermore, enhanced CAT activity in duckweed was suggested as a protective strategy adopted by this plant under DEHP stress (Xu et al., 2010). The current results are in agreement with the previous observations. These results suggested that coordination of antioxidants is needed for the detoxification mechanism underlying stress in plants.

Since peroxidases play a pivotal role in plant growth and development (Passardi et al., 2005). They are involved in lignin biosynthesis and consume H_2_O_2_. Meanwhile, guaiacol peroxidases (GPX) activity in this study exhibited a significant decline at lower concentrations upto 40 ppm and then enhanced at higher concentrations of DEP, forming a U-shaped pattern (Fig. 2). This explained the efficiency of the plant to show up-regulation of both the peroxidases (APX and GPX) to cause a diminution of H_2_O_2_ when subjected to DEP-stress. Increased GPX activity was also observed in *Hordeum vulgare* exposed to BBP for seven days (Kumari & Kaur, 2019). Present results displaying an increasing trend in GPX activity are further supported by the observation of Zhang et al. (2015) on cucumber seedlings under DMP stress.

Moreover, the activity glutathione reductase (GR) enzyme which catalyzes the reduction of oxidized glutathione (GSSG) to reduced glutathione (GSH) in the presence of NADPH, thus, maintain a GSH/GSSG ratio (Apel & Hirt, 2004). During stress, conserved disulphide bridge in the structure of GR easily breaks. GR activity was also also found to be elevated in this study (Fig. 2). Results are in coherence with the earlier reports on plants (Wang et al., 2008; Zhang et al., 2014). Hence, enhanced enzymatic activities paralleled the accumulation of MDA and ROS in fronds of *S. polyrhiza* indicating the response of this plant to oxidative stress. Cheng (2012) reported a 1.79 fold increase in GR activity of duckweed under DEP exposure for four days. Thus, present data revealed significant enhancement in enzymatic activities, implying efficient ROS scavenging during seven days of culture.

Additionally, toxicological implications faced by *S. polyrhiza* under DEP stress were revealed by viewing electron micrographs of ventral surface of DEP stressed fronds which showed many closed stomata as compared to unstressed fronds (Fig. 3). Stomata are responsible for gaseous and water vapours exchange between plants and the surrounding environment. It is plausible that *S. polyrhiza* attained DEP stress tolerance attributes by showing alterations in stomatal movements. Accumulation of abscisic acid during stressful conditions is also one of the reasons for the closure of stomata (Zhang & Outlaw Jr, 2001).

Also, confocal micrographs depicted no or less fluorescence in the roots of control plants, while high fluorescence was observed in DEP treated roots when treated with fluorescent dyes (Fig. 4). Loss of integrity in the plasma membrane was quite evident from the confocal micrographs of propidium iodide treated roots, as this dye is unable to penetrate an intact cell. This indicated that *S. polyrhiza* responded to DEP stress and fluorescence helps in determining apoptosis. Treatment with DCFDA and MCB fluorescent dyes revealed the generation of ROS and elevation of GSH levels in treated samples.

## Conclusions and Future Prospects

From the present investigation, it can be inferred that most phytotoxic effects were prominent at the highest tested concentration (400 ppm) of DEP. Decrement in photosynthetic pigments, protein and carbohydrate content were positively correlated with increment in the dose of DEP. Moreover, DEP accumulation in plants also triggered oxidative stress evident from increased MDA, proline, phenol content and leakage of ions, thus implying elevated levels of ROS. To cope up with DEP mediated toxicity, the chain of antioxidative enzymes (SOD, CAT, APX, GPX and GR) get activated, demonstrating how well plant efficiently defends itself against DEP toxic effects. All the biochemical parameters and the antioxidant enzymatic activities that were tested here will be used as an indicator in examining adverse effects at varying doses of DEP in aquatic plants. Further, a proper understanding of the alterations in biochemical activities and adaptive mechanisms shown by *S. polyrhiza* under DEP stress will give a baseline idea in developing the phytoremediation ability of plants in the future. From the work presented here, this aquatic plant can be effective as a biosorbent for the removal or alleviation of low level of DEP. Association of *S. polyrhiza* with various plant growth-promoting rhizobacteria (PGPR) can also be utilized as an effective strategy to enhance the removal of phthalates. Findings will further contribute to the evaluation of environmental risks posed by DEP in the aquatic ecosystem. However, the binding or interaction of DEP with plasma membrane, mechanism of its uptake and sequestration in plant or detoxification pathways adopted by the plants need to be thoroughly explored.

##  Supplemental Information

10.7717/peerj.8267/supp-1 Dataset S1 Accumulation of diethyl phthalate (DEP) by *S. polyrhiza.*

10.7717/peerj.8267/supp-2 Dataset S2 Effect of DEP stress on antioxidant enzymatic activities of *S. polyrhiza*

10.7717/peerj.8267/supp-3 Dataset S3 Effect of diethyl phthalate (DEP) on growth parameters of *S. polyrhiza.*

10.7717/peerj.8267/supp-4 Dataset S4 Effect of diethyl phthalate (DEP) on chl a, chl b, total chlorophyll, carotenoids and anthocyanin pigment in *S. polyrhiza*

10.7717/peerj.8267/supp-5 Dataset S5 Effect of diethyl phthalate (DEP) on carbohydrate, protein, MDA, total phenolic content and electrolyte leakage in *S. polyrhiza*

## Additional Information and Declarations

### Data Availability

The following information was supplied regarding data availability: The raw measurements are available in the Supplemental Files.
